# Core as a Novel Viral Target for Hepatitis C Drugs

**DOI:** 10.3390/v2081734

**Published:** 2010-08-20

**Authors:** Arthur Donny Strosberg, Smitha Kota, Virginia Takahashi, John K. Snyder, Guillaume Mousseau

**Affiliations:** 1 Department of Infectology, The Scripps Research Institute-Scripps Florida, 130 Scripps Way, Jupiter, FL-33458, USA; E-Mails: smitha@scripps.edu (S.K.); vtakahas@scripps.edu (V.T.); mousseau@scripps.edu (G.M.); 2 Department of Chemistry, The Center for Chemical Methodology and Library Development, Boston University, Boston, MA 02215, USA; E-Mail: jsnyder@bu.edu

**Keywords:** Hepatitis C Virus, HCV, core dimerization, peptide inhibitors, small molecule inhibitors, virus assembly

## Abstract

Hepatitis C virus (HCV) infects over 130 million people worldwide and is a major cause of liver disease. No vaccine is available. Novel specific drugs for HCV are urgently required, since the standard-of-care treatment of pegylated interferon combined with ribavirin is poorly tolerated and cures less than half of the treated patients. Promising, effective direct-acting drugs currently in the clinic have been described for three of the ten potential HCV target proteins: NS3/NS4A protease, NS5B polymerase and NS5A, a regulatory phosphoprotein. We here present core, the viral capsid protein, as another attractive, non-enzymatic target, against which a new class of anti-HCV drugs can be raised. Core plays a major role in the virion’s formation, and interacts with several cellular proteins, some of which are involved in host defense mechanisms against the virus. This most conserved of all HCV proteins requires oligomerization to function as the organizer of viral particle assembly. Using core dimerization as the basis of transfer-of-energy screening assays, peptides and small molecules were identified which not only inhibit core-core interaction, but also block viral production in cell culture. Initial chemical optimization resulted in compounds active in single digit micromolar concentrations. Core inhibitors could be used in combination with other HCV drugs in order to provide novel treatments of Hepatitis C.

## 1. Introduction

Hepatitis C virus (HCV) infects over 130 million people world-wide [1–2]. It is a single stranded positive RNA enveloped virus which belongs to the hepacivirus genus in the flaviviridae family [3–6]. The 9.6 kilobase long viral RNA encodes a polyprotein of approximately 3,010 amino acid residues [7]. The HCV polyprotein is co- and post-translationally processed into ten individual components by host cell signal peptidases and viral peptidases [8]. All ten proteins are essential for viral infectivity, and most are endowed with several functions [7,9].

HCV is the main cause of chronic liver disease in humans. There is no anti-HCV vaccine. The only effective 48-week treatment currently available combines pegylated interferon-alpha and ribavirin, but the overall success rate is less than 50%, depending on the viral genotype and strain [10]. Its use is plagued with side effects and poor compliance [11]. Small molecule HCV-specific inhibitors that target directly the individual viral proteins NS3/NS4A serine protease, and RNA-dependent RNA NS5B polymerase are in advanced stages of clinical development. [12–15]

Here we discuss core, the HCV capsid protein, as a novel non-enzymatic target for anti-HCV drug discovery likely to yield agents with a resistance profile different from inhibitors of HCV enzymes. We review core’s role in the assembly of the virion, and we describe assays to identify, select and optimize peptides and small molecule inhibitors of core dimerization and viral production. We discuss the use of such inhibitors to probe and control core’s activity in HCV.

## 2. Core as a target protein for anti-HCV drug discovery

Viral capsid proteins have been proposed previously as targets for anti-viral drugs, based on the fact that they are essential for virus assembly, are highly conserved even in viruses which mutate extensively, and self-assemble in well-controlled conditions easy to assay *in vitro* [16]. Despite these advantages, only Hepatitis B and Human Immunodeficiency Viruses have so far provided good examples that support the validity of the strategy [17–19]. What makes HCV core an especially attractive target, in addition to its dual role in viral infection and persistence, is the fact that it is the most conserved of all HCV proteins, across the 6 major genotypes, and that it is the least variable of the ten HCV proteins in variant viruses emerging constantly in patients [10]. This exceptional level of conservation reflects its essential role and suggests that its use as a therapeutic target across all genotypes is unlikely to be affected by mutations causing resistance, thus providing a profile quite distinct from other direct-acting drugs. While mutations in core influencing HCV’s response to interferon have been studied recently in connection with treatment with a new anti-protease inhibitor [20], such substitutions remain exceedingly rare, when compared to the multiple mutations emerging in NS3 and NS5 enzymes, mostly used so far as targets for anti-HCV drug discovery [21–22]. Finally, adding to these advantages, biochemically functional C-terminally truncated versions of core are easy to prepare and purify, and readily dimerize and oligomerize *in vitro* in absence or presence of RNA [23].

## 3. Core’s role in HCV’s life cycle

### 3.1. Core interactions with other HCV proteins

Core is essential for nucleocapsid assembly and interacts with several other viral proteins, namely the E1 glycoproteins [24], p7 and NS2 [25], NS3 [26] and NS5A [27]. These interactions were confirmed by immuno-staining followed by confocal microscopy which revealed co-localization of core with NS5A and NS3 on lipid droplets [26,28] and were supported by yeast-two hybrid analyses [29–31] and co-precipitation data [28,32]. Molecular genetics provided additional evidence for core-NS protein interactions: spontaneous mutations in p7 and NS2 rescued production of virus mutated in core [25]; site-directed mutagenesis, alanine scanning [25], and other methods led to the identification of several residues in both core and NS5A presumably involved in the co-localization of the two proteins, although direct evidence for *in vitro* binding of NS5A to core has proven to be difficult to obtain [32–33].

### 3.2. Core’s role in assembly

Core, the capsid protein, plays a central role in the HCV life cycle: it is essential for lipid droplet mobilization [34–35], recruitment of HCV replicase proteins, nucleocapsid formation, and assembly and release of viral particles from infected cells [36–37].

**Figure 1.**
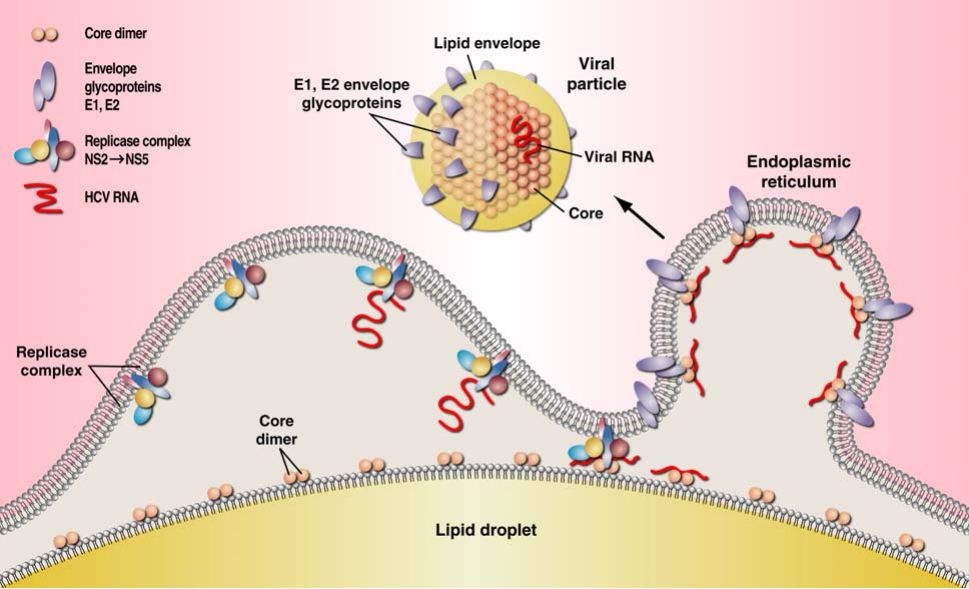
A schematic (left to right) model for assembly of the HCV particle. Core-directed assembly takes place at the surface of lipid droplets (“LD”). Core, originally made as a 191 residue protein, migrates from the endoplasmic reticulum to the surface of LD’s after removal of the C-terminal 14 residues by a host signal peptide peptidase [38–39,46]. Core oligomers recruit to the LD surface NS3, NS5A, NS5B, and possibly other HCV replicase proteins implicated in the synthesis of HCV RNA, including p7 and NS2 [4,27,47]. HCV nucleocapsids resulting from the binding of RNA to core are then associated via unknown processes with HCV glycoproteins E1 and E2 and processed through the VLDL assembly pathway [27,48] (not shown here), to yield lipid encapsulation, resulting in budding of viral particles [49]. Whether the replicase complex as such interacts with core before or after RNA is made, and whether RNA binds to core dimer / oligomer on LD’s is not yet known, nor is the stoechiometry between core, RNA and the E1, E2 glycoproteins (modified from [28]).

The sequence of events leading to core-orchestrated HCV particle assembly is schematically depicted in Figure 1, and can be described to progress from left to right as follows: after translation, the HCV polyprotein is directed to the Endoplasmic Reticulum (“ER”) by a signal peptide sequence situated at the C-terminal end of core, immediately adjacent to the E1 glycoprotein. Two successive cleavages, first by a cellular signal peptidase [38] then by a cellular signal peptide peptidase [39–40] result respectively in release from the polyprotein and migration of mature, probably dimerized /oligomerized core, to the surface of LD’s [41]. Core then recruits, most if not all non-structural HCV proteins from the ER: NS3 [26], NS5A [28,32], NS5B and possibly p7 and NS2 [42–43], which together constitute the replicase complex, responsible for RNA replication (Figure 1). At the surface of LD’s, core also interacts with a number of non-HCV proteins (not represented in Figure 1) , namely those involved in Very Low Density Lipoproteins (VLDL) biogenesis such as ApoE, ApoB and Microsomal Transfer Protein [44–45]. Newly synthesized HCV RNA is transferred from the replicase complex to core, and the resulting nucleocapsid is associated into lipid-encapsulated particles, together with E1 and E2 glycoproteins (Figure 1). The stoechiometry, the sequence and timing of these events are still debated.

## 4. Core’s structure and function

**Figure 2.**
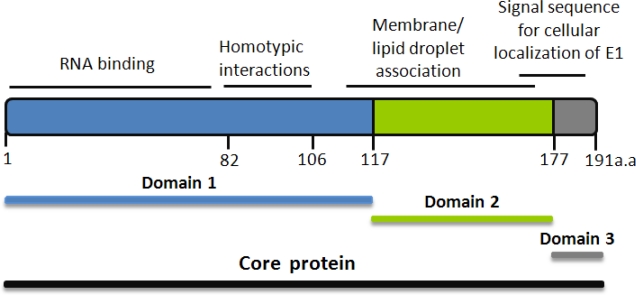
Structural and Functional domains of the HCV core protein. Core is made as a 191 residue long polypeptide, and is trimmed by a signal peptide peptidase, down to a 177-residue mature protein [46,57]. Various synthetic C-terminally truncated versions of recombinant core have been used in order to characterize its functional domains: core82 [59], core106 [23], core122, “D1” [55], core169 [55], and core177, “D2” [39,57].

Core contains a number of amino-acid residues essential for assembly and release of the viral particle [50], and for interaction with other HCV proteins, such as the E1 glycoprotein and replicase proteins NS3 [26] and NS5A [51]. These various roles have been assigned to different parts of the protein (Figure 2).The N-terminal basic domain, up to residue 117 (“D1”) binds and promotes dimerization of the viral RNA [52], leading to formation of viral nucleocapsid [53–54]. Recent analysis of core mutants in this domain revealed that D1 also contains a sequence crucial for core envelopment by endosomal membranes [41]. The generally more hydrophobic C-terminal domains “D2”, and “D3”, interact with the Lipid Droplets, with the E1 and E2 glycoproteins, and with several host proteins themselves involved in lipid transport [27,47,55]. The 177 residue mature core [46,56] is translocated from the endoplasmic reticulum to the lipid droplet surface [57]: the transmembrane C-terminal core peptide remains in the ER, where it serves as the E1 glycoprotein signal sequence [39,58]. Core’s role as a powerful chaperone, and as an inducer of dimerization of newly-made HCV RNA has been well recognized [53]. It’s essential function is increasingly appreciated as an organizer of particle assembly (Figure 1) as well as mediator of host-pathogen interactions.

Dimerization and most interactions with intracellular proteins are mostly mediated by the N-terminal region of core, whereas binding to cellular membranes [60], to LD’s and to glycoprotein E1 have been mapped to the more hydrophobic C terminus [54]. The N-terminal half of core contains many of the residues essential for homotypic interaction and nucleocapsid formation [61] [29,62–63]. In a cell-free system for core synthesis, the residues critical for capsid formation were all located in the first 68 positions of core [64] and core 1 to 82 was designated as the minimal domain for nucleocapsid assembly [61,65]. Alanine scanning revealed numerous core residues essential for infectious virus production, including a significant number in the first 120 positions of the protein [25]. The C-terminal region comprised between residues 118 and 169 is required for proper folding of the whole core protein and preservation of interactions not observed with D1 [55]. For instance, mutations of residues at position 130, 138 or 143 all abrogate association with LD’s and virus production [34,66]. Core’s D2 C-terminal half thus harbors several sites that are essential for the protein’s roles in HCV’s life cycle, e.g. for binding and attracting other HCV proteins to LD’s. In addition, core’s C-terminal is involved in mechanisms of viral persistence, oncogenesis [67] and steatosis [68].

## 5. Assays for analyzing core function:

To improve the understanding of core oligomerization and the role of dimerization in interaction with other proteins and nucleocapsid formation, various biochemical assays were developed:

### a. Assays for Nucleocapsid formation

Several groups have proposed assays to evaluate the function of core based on its capacity to oligomerize or form nucleocapsid-like structures in the presence of RNA [59,61,64–65]. An assay for measuring the turbidity resulting from the addition of RNA to core served to define core-derived peptides essential for this complex formation [61]. Unexpectedly, these peptides did not originate from the core homotypic region, between residues 82–102, thought to be responsible for core dimerization. Furthermore, the nucleocapsid assay did not distinguish between RNA from HCV or from a variety of other sources, including tRNA.

### b. Core dimerization

In order to physico-chemically characterize core dimerization and identify identify high affinity inhibitors, three types of assays were designed and developed: Enzyme Linked Immuno Sero Assay (“ELISA”), Time Resolved-Fluorescence Resonance Energy Transfer (“TR-FRET”) and Amplified Luminescent Proximity Homogeneous Assay (AlphaScreen) [23,69–70].

To avoid complications due to limited solubility of mature (ie: D1+D2) core protein, these assays were developed using a D1-like dimerizing portion of core, comprising the first 106 residues, and further designated as “core106”.

**Figure 3.**
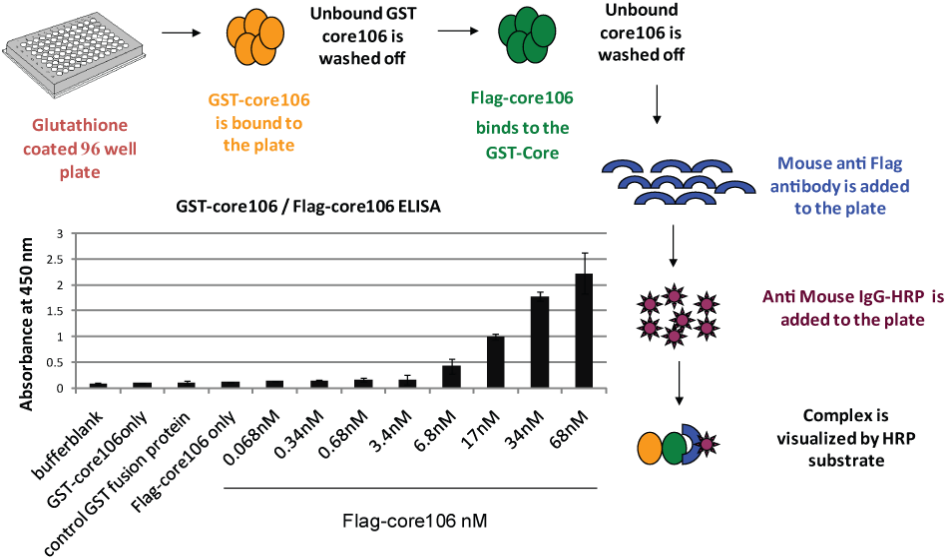
A scheme and data for sandwich ELISA showing dimerization of core106. Core106 was tagged with either a “GST” tag or a “Flag” tag. A sandwich ELISA was performed by adsorbing GST-core106 on a Glutathione-coated plate and different concentrations of Flag-core106 were added to the plate. Mouse-anti-Flag antibody, horse radish peroxidase tagged anti-mouse IgG, and Ultra TMB-HRP, a horse radish peroxidase substrate were utilized for the visualization of GST-core106 and Flag-core106 complex.

**Figure 4.**
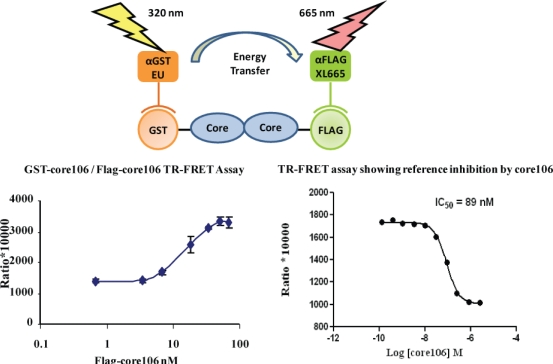
A scheme and data for TR-FRET assay for monitoring dimerization of core106. Core106 was tagged with either a “GST” tag or a “Flag” tag. Europium cryptate-tagged anti-GST and Allophycocyanin (XL-665)-tagged anti-Flag antibodies were used to measure the core-core interaction. Since no known inhibitor of core dimerization was available, untagged core106 domain was used as a reference inhibitor. Top panel – Scheme for core106 TR-FRET assay. Bottom left panel- Dose response of GST-core106 and Flag-core106 dimerization. Bottom right panel – Inhibition of GST-core106 and Flag-core106 dimerization by reference inhibitor core106. The transfer of fluorescence was measured as a ratio of (signal at 665nm / signal at 620nm) *10,000.

**Figure 5.**
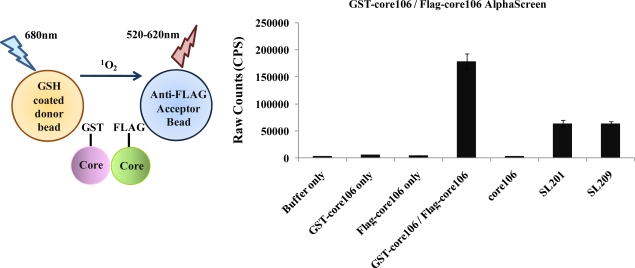
Left panel – Scheme for core106 AlphaScreen assay. This assay is based on the use of photoactive donor and acceptor beads that recognize specific tags on interacting proteins. An interaction between proteins brings the beads in close proximity. This triggers a cascade of chemical reactions which produces a greatly amplified signal. When excited, a photosensitizer in the donor bead converts ambient oxygen to a more excited singlet state which then reacts with a chemiluminescent molecule in the acceptor bead which further activates fluorophores contained within the same bead to emit light [71]. Right panel-Assay confirming interaction of GST-core106 and Flag-core106 and inhibition of the interaction by untagged core106 [23,69].

1. For developing a sandwich ELISA, GST-tagged core106 domain was adsorbed on microplates coated with glutathione, and a horse-radish peroxidase goat anti-mouse antibody was used to demonstrate binding of anti-Flag antibody to Flag-tagged core106 itself bound to GST-core106 (Figure 3). This result demonstrated hetero-dimerization of the two fusion proteins. Free GST, Flag peptide or untagged core106 each displaced specific binding to background levels. [23].
2. The ELISA was useful to qualitatively demonstrate the interaction when using alternately tagged core106 fusion proteins, but the need for multiple washes hampered a precise analysis of the interaction, and precluded its use for screening of large compound libraries. For this purpose, a TR-FRET homogenous assay was developed, using the same GST-and Flag-tagged core106 proteins (Figure 4) [23,69]. Fluorophore-labeled antibodies against the GST and Flag tags allowed the authors to quantitatively measure the formation of dimers and oligomers of core106. The TR-FRET was particularly well adapted for robotized high-throughput screening in 384- and 1,536-well microplates of large small compound libraries [69]. The typical signal to background ratio shown in Figure 4 left, was rather low, although the inhibitory signal (Figure 4 right) was quite adequate for identifying “primary hit” inhibitors [69].
3. To develop a more sensitive assay for precise dose-response analyses, an AlphaScreen assay was developed, as a secondary format for monitoring core106 dimerization, but using donor and acceptor beads coated respectively with Glutathione or anti-Flag antibodies respectively, resulting in assays with at least 50-fold signal to background ratios (Figure 5) [23,70]. Again, untagged core106 domain was used as a reference inhibitor, with an IC_50_ of 89 nM [23]. While AlphaScreen beads are not easily handled by most robotic suites found in high-throughput screening laboratories, and sometimes vary in day-to-day stability, they provided a remarkably sensitive format for following chemical optimization of primary hit compounds first identified in large scale screening [70].

## 6. Inhibitors of core dimerization

### 6.1. Inhibition of core106 and core169 dimerization by peptides

Protein-protein interactions can generally be inhibited by peptides derived from either one of the two partners [72].To identify such inhibitor peptides of core dimerization, fourteen 18-residue peptides derived from core were evaluated in energy transfer TR-FRET and AlphaScreen assays validated by dose-response analyses [23,69–70]. Two peptides SL173 and SL174, overlapping by 11 residues, inhibited core dimerization by 68 and 63% respectively. A third peptide, SL175, corresponding to SL174 C-terminally truncated by three residues, inhibited core dimerization by 50%. These three inhibitory peptides share the sequence:

-Gly-Trp-Ala-Gly-Trp-Leu-Leu-Ser-Pro-Arg-Gly-

which corresponds to a “hot spot” defined as a small subset of residues involved in protein-protein interactions that contributed most of the free energy of binding between the two protein partners [72].

Peptide SL200, containing only this 11-residue sequence, inhibited core dimerization by 30–40%, but was not soluble enough to reach concentrations that would yield higher inhibition rates [23]. The Ser residue within SL200 corresponds to Ser99 which was earlier reported to be essential for core function in the whole virus: its substitution by an Ala residue strongly affects HCV viability [25], and reduces fourfold its ability to inhibit core dimerization [23]. The SL200 sequence is conserved among genotypes 1a, 1b 2a and 3, and is situated within the 82–102 homotypic domain of core defined earlier [73]. A dose-response analysis of peptide SL175 yielded an IC_50_ of 21.9 μM. SL571, a control peptide which contains the reverse sequence of SL175 displayed no effect on core dimerization [23].

Direct and specific interaction of SL175 with core was demonstrated by two physico-chemical methods. For Fluorescence Polarization (“FP”) studies, SL175 was conjugated to fluorophore Alexa488 and a dose-response analysis yielded a Kd of 1.9 μM for the binding of the coupled peptide to core106. Displacement of the A488 peptide by free peptide yielded an IC_50_ of 18.7 μM, in close agreement with the value of 21.9 μM obtained by AlphaScreen. Surface Plasmon Resonance (“SPR”) analysis done with core169, yielded an apparent Kd of 7.2 μM [23].

Cross-linking studies performed as described [23], confirmed that SL175 binds directly to core106 monomers and dimers [74]. Control peptide SL571 displayed no evidence of binding to either core truncated protein, in any of the FP, SPR or cross-linking studies [23].

### 6.2. Inhibitors of core dimerization by organic molecules

The TR-FRET and AlphaScreen assays described in section 5 were used to identify organic molecule inhibitors of core dimerization in a library of 2,240 compounds from the Center for Chemical Methodology and Library Development of Boston University [69–70]. Included in this collection were representatives of a small molecule, 132-membered library of tetracyclic heterocyclic compounds. Compounds with this general chemotype were originally viewed as desirable due to their structural similarity to the Aspidosperma alkaloids, well known for their broad range of biological activities [75–76]. Furthermore, their production was relatively straightforward and easily amenable to parallel synthesis protocols [77].

**Figure 6.**
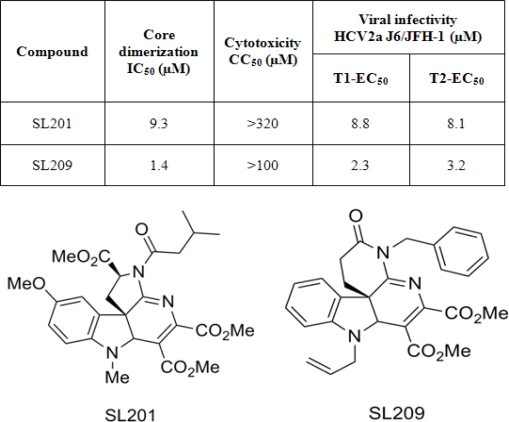
Structure and activity of small molecule inhibitors of HCV core dimerization and virus production [69–70]. Top: half maximal inhibitory concentration (IC_50_) values determined in the core dimerization assay. The half maximal cytotoxicity concentration (CC_50_) values (see section 7) were determined using uninfected hepatoma cells. The T1 and T2 half maximal effective concentration (EC_50_) values were obtained using the same cells but infected with HCV 2a J6/JFH1 virus at two different passages [23,69–70]. Bottom: chemical structures of SL201 and SL209 [69].

The best “primary hit” compound present in this indoline alkaloid-type library, designated as SL201 (MW 513 daltons, structure in Figure 6 top), was shown to block core106-core106 and core106-core169 dimerization by over 90%, nearly as efficiently as core106 itself, used as a 100% control inhibitor. A dose–response analysis yielded an IC_50_ equal to 9 μM [69].

Using SL201’s structure as prototype, a second generation library of 82 members was prepared, exploiting chemistry similar to that used in preparing SL 201. The scaffold of this library varied the D-ring of the initial scaffold, featuring a δ-lactam with a diversified lactam nitrogen (Figure 6 bottom). Forty-eight of these new analogues were screened, and those capable of inhibiting over 50% of the GST-core106/Flag-core106 interaction were further investigated by dose-response analysis yielding IC_50_ values ranging from 20 down to 1.4 μM. SL209 was the most promising compound in that second series, and its structure is also presented in Figure 6 [70]. The activities of the members of this second generation library established that the D-ring ester group of the first generation was not essential for the activity of SL 201 [69–70].

## 7. Inhibition of HCV production by inhibitors of core dimerization

Huh-7.5 hepatoma cells were electroporated with RNA from HCV and the 72-hour supernatant was used as a source of infectious virus. This supernatant was combined with peptides or compounds identified as inhibitors of core dimerization [23,70]. Two stages of infection were studied: T1, corresponding to the initial 72-hour culture of naïve cells infected in the presence of the inhibitor, and T2, corresponding to the second 72-hour passage through naïve cells. HCV RNA detected in T1 results from actual viral infection and replication. HCV RNA detected in T2 results from infectious virus secreted in T1 and replicated in freshly infected naïve cells.

After determining that inhibitors of core dimerization were not toxic for Huh-7.5 hepatoma cells, their capacity to inhibit production of HCV by *in vitro* infected cells was evaluated [23,69–70], using the HCV 2a J6/JFH-1 culture system [78–80].

### 7.1. Peptide inhibitors of HCV

Peptides SL173, 174 and 175 displayed no significant toxicity towards uninfected cells, when used below 100 μM concentrations [23]. When added in 10 to 20 μM concentrations to HCV-infected cells, these peptides reduced production of the virus, as determined both by limited dilution Tissue Culture Infectious Dose – 50% (TCID-50) studies [23] and by Real Time RT-PCR measurement of HCV RNA[69–70].

For peptide SL173, this result was indirectly confirmed by Cheng *et al.* [81] who showed by direct assay of the 441 HCV polyprotein-derived peptides added one by one to HCV-infected cells, that a core-derived peptide identical to SL173, reduced respectively by a factor of 9 and 11 the production of HCV RNA in 24-hour and 72-hour culture [81].

### 7.2. Small compound inhibitors of HCV

Compounds SL201 and SL209 which did not display toxicity up to 320 μM and 100 μM respectively (Figure 6) were evaluated for inhibition of HCV production in culture at two stages, T1 and T2. HCV RNA was quantified by Real-Time RT-PCR. Compounds SL201 and SL209 reduced HCV RNA production respectively by about two logs with EC_50_ of 8.8 μM for T1 and 8.1 for T2, and 2.3 μM for T1 and 3.2 μM for T2 (Figure 6) [69–70].

### 7.3. Mode of action of inhibitors

#### 7.3.1. Peptides

To investigate their mode of action, inhibitor peptides were added before, together and after infection with HCV. Reduction of infectivity was only observed after infection was established [23], suggesting that the peptides had no effect on entry or replication, but might act on assembly. This conclusion would fit with the fact that the peptides were originally identified as inhibitors of core dimerization. No evidence was reported however to demonstrate that the peptides were actually able to penetrate the HCV-infected cells. Another 18-mer peptide [8] derived from the same NIH library of 441 HCV polyprotein-derived peptides, was detected inside HCV-infected cells, after coupling with a dansyl moiety, which served as an epitope for anti-dansyl antibodies [81]. Although that peptide had a different sequence and very different biological properties from SL173 and SL174, its similar size suggests that 18-mer peptides can indeed penetrate Huh-7.5 HCV infected cells.

#### 7.3.2. Small organic molecule inhibitors

The mode of action by which organic molecules SL201 and SL209 reduce HCV infectivity has not yet been established. It was shown however that the compounds had no effect on production of HCV replicons, confirming that they do not affect HCV RNA replication [69–70]. The inhibitors appeared to act on virus production by cells three days after electroporation with RNA from HCV: this experiment was actually done for SL201 on RNA from HCV of two distinct strains: J6/JFH-1 of genotype 2a [78–80] and CG of genotype 1b [82]. The inhibitor acted equally well on the 2a virus released in the supernatant of cells infected by T1 or T2 [23,69]. These results suggest that SL201 affect neither virus entry into the cells, nor replication of RNA, supporting the hypothesis that this inhibitor of core dimerization / oligomerization might act on virion assembly.

## 8. Future developments

Core plays an important role in various steps of the HCV infectious cycle including nucleocapsid assembly and recruitment of other HCV proteins to the site of viral production. The precise understanding of the importance of core oligomerization and of its interactions with other HCV and cellular proteins will provide unique insights in its role in the viral life cycle. Peptides and small molecule disruptors of core dimerization, which act as potent inhibitors of HCV, constitute unique novel molecular probes for HCV function and complement current molecular genetics approaches for the study the HCV life cycle. Importantly, these studies might also impact studies of other Flaviviridae viruses for which coupling between virion assembly and genome replication has been suggested to result from interaction between core and non-structural proteins. Furthermore, future drugs acting on core as a target might potentially provide an important breakthrough for novel combination treatments of Hepatitis C.
